# The Use of the Moustache and Beard in the Royal Navy

**Published:** 1859-01

**Authors:** Daniel John Duigan

**Affiliations:** Surgeon, *R.N.*


### ON THE USE OF THE MOUSTACHE AND BEARD IN THE ROYAL NAVY.

By DANIEL JOHN DUIGAN, M.D., F.R.C.S.I., Surgeon, *R.N.*

DURING the late war with Russia the English soldier was permitted to wear his beard and moustache—the marks of manhood, furnished by nature to some wise end.

The seamen who co-operated with the troops, under the appellation of the Naval Brigade, followed the example of the soldiers. Since the war, the army has been suffered to retain the beard and moustache (under certain regulations), whilst the navy has been compelled to resume the use of the razor. On physiological grounds, I beg to recommend the growth of facial hair, more especially for the seamen and marines (including the officers) serving in Her Majesty's navy. The moustache and beard protect a man from the inhalation of dust, from solar blistering, from diseases of the respiratory organs, and from malarious influence. I do not assert that they serve these good purposes absolutely, but they give most efficient aid.

(*a*). Facial hair protects the air-passages from dust.

Now that the steam-engine has come into general use in the ships of the Royal Navy, this is a subject of interest to every man in the service, and especially to the engineers and stokers. The operation of saturating the ashes with water, causes a cloud of impalpable dust and steam, which envelopes the men engaged in the duty.

"Trimming coal," and "coaling the ship," are attended by much dust; and tube-cleaning is distressing to the men to such a degree, as to cause them to wear a piece of bunting over the mouth as a respirator.

In addition to coal-dust the seamen are exposed, on certain stations, to an impalpable powder conveyed in the air out to sea for a considerable distance from the land. This is the case on the Mediterranean station, at Malta, and in the vicinity of the Egyptian shore. On the West African station, too, the same evil is experienced. Dr. Bryson* writes respecting the Bay of Portendico—

"In this locality, from its proximity to the great Desert of Sahara, the harmettan is more severely felt than upon any other part of the station. It chiefly prevails during the months of December, January, and February; from its ex-

* See "Report on the Climate and Principal Diseases of the African Station".

treme dryness, it has the effect of imparting to the mucous membrane of the lips, nose, and eyes, the sensation of being parched. The first two blister and crack, while the last become inflamed. . . . But the most remarkable phenomenon attending it, is the quantity of impalpable sand that it brings from the desert, which, from its wonderful subtilty, penetrates even the smallest crevice. . . . Every person exposed on deck for a few hours is so covered with dust, that he presents more the appearance of a miller than of a seafaring man."

Another source of dust on shipboard is the dry holystoning of the decks.

In all these instances the moustache opposes a barrier to the entrance of the dust, and thus renders good service.

(*b*). Facial hair is protective against solar blistering.

Blistering of the skin is a common effect of exposure to the sun's rays in hot climates. This annoying effect of heat is mitigated by a hairy covering to the skin.

(*c*). Facial hair protects the respiratory organs.

The first indication of a damp atmosphere is the moisture upon the moustache, due to the hygrometric quality of hair. The wearer of the moustache derives very great comfort from nature's respirator, for the moisture trickles away by the side of the mouth in lieu of entering the air passages. The hairy respirator removes the water from the air, and acts thus as a desiccating body. The natural heat of the surface of the face and throat are kept up by the beard, and the individual possessing this noble ornament escapes from a long list of inflammatory affections, amongst which I may enumerate soreness of the throat, catarrh, bronchitis, and herpes labialis. He is also saved from the torture of pains in the jaws, due to neuralgia and rheumatism. I could mention other maladies from which the beard affords protection; but I forbear, lest I be thought to exaggerate on this subject. The exemption of the Naval Brigade in the Crimea from diseases of the respiratory organs was most remarkable. One officer shaved off his moustache (which was thick and bushy), on rejoining his ship. The immediate result was an attack of pulmonary inflammation, which well nigh cost him his life.

It is a common practice to apply a flannel roller to the throat when it is sore; the beard is nature's prophylactic roller, better than flannel after the cold has been caught.

(*d*). The moustache has been regarded by some writers as protective against the influence of malaria.

It is highly probable that a hairy covering to the mouth and throat may act in this way by sustaining the warmth of the parts, and by rendering the inspired air dry.

There are observations to show that malarious influence can be broken in ways that are inexplicable, such as by a belt of trees, by a running stream, and such like. A gauze curtain is said to afford immunity at Padua. If such be the fact, a respirator ought equally to afford protection. The shepherds dwelling in the Campagna di Roma were formerly swept away in numbers by malarious fevers. Now, they are comparatively safe by attention to the laws of hygiene, namely, the erection of their dwellings at a high level, the use of flannel clothing, and the growth of their beards and moustachios.

In conclusion, I would observe that, apart from the sanitary view of the question, shaving is to the sailor a cruel operation. Circumstances are unfavourable to him. Taking them *seriatim*, they are as follows :—1. Blunt razor ; 2. Cold water ; 3. Scanty supply of water ; 4. Darkness, or insufficient artificial light ; 5. Fragment of a looking glass (in consequence of previous breakage) ; 6. Rolling and pitching ship ; 7. Stiffness of face from exposure to cold air on the morning watch ; 8. Ill temper at being forced to shave.

---

